# Supplementary material for: Preoperative versus Postoperative Compensation of the Contralateral Normal Kidney in Patients Treated with Radical Nephrectomy for Renal Cell Carcinoma
Source: J Clin Med. 2021 Oct 24;10(21):4918. doi: 10.3390/jcm10214918 (PMC8584614; doi:10.3390/jcm10214918)
Supplement: Supplementary file 1 [file jcm-10-04918-s001.zip › jcm-1405960-supplementary.pdf]

Supplementary Online Contents

Table S1. Univariate logistic regression analysis of factors associated with compensation from preoperative period to postoperative 1 week (process 1) in patients treated with radical nephrectomy for RCC

Table S2. Univariate logistic regression analysis of factors associated with compensation from postoperative 1 week to postoperative 5 years (process 2) in patients treated with radical nephrectomy for RCC

Table S3. Univariate logistic regression analysis of factors associated with compensation from preoperative period to postoperative 5 years (process 1+2) in patients treated with radical nephrectomy for RCC

Table S1. Univariate logistic regression analysis of factors associated with compensation from preoperative period to postoperative 1 week (process 1) in patients treated with radical nephrectomy for RCC

	Univariate	
	**HR (95% CI)**	***p***
Sex		
Male	Reference	
Female	1.509 (1.096-2.077)	0.012
Age	1.026 (1.012-1.041)	<0.001
BMI	0.956 (0.910-1.004)	0.072
DM		
No	Reference	
Yes	1.236 (0.795-1.921)	0.347
HTN		
No	Reference	
Yes	1.187 (0.849-1.659)	0.317
Preop. GFR	0.942 (0.932-0.952)	<0.001
Tumor volume	1.005 (1.004-1.006)	<0.001
CNK volume	1.007 (1.003-1.011)	0.001

HR, Hazard ratio; CI, Confidence interval; BMI, Body mass index; DM, Diabetes mellitus; HTN, Hypertension; Preop., Preoperative; GFR, Glomerular filtration rate; CNK, Contralateral normal kidney.

Table S2. Univariate logistic regression analysis of factors associated with compensation from postoperative 1 week to postoperative 5 years (process 2) in patients treated with radical nephrectomy for RCC

	**Univariate**	
	**HR (95% CI)**	***p***
Sex		
Male	Refence	
Female	0.680 (0.458-1.010)	0.056
Age	0.980 (0.964-0.996)	0.016
BMI	1.111 (1.049-1.176)	<0.001
DM		
No	Reference	
Yes	0.414 (0.208-0.824)	0.012
HTN		
No	Reference	
Yes	0.798 (0.526-1.209)	0.287
Preop. GFR	1.000 (0.991-1.010)	0.964
Tumor volume	1.000 (0.999-1.002)	0.601
CNK volume	1.001 (0.996-1.006)	0.778

HR, Hazard ratio; CI, Confidence interval; BMI, Body mass index; DM, Diabetes mellitus; HTN, Hypertension; Preop., Preoperative; GFR, Glomerular filtration rate; CNK, Contralateral normal kidney.

Table S3. Univariate logistic regression analysis of factors associated with compensation from preoperative period to postoperative 5 years (process 1+2) in patients treated with radical nephrectomy for RCC

	**Univariate**	
	**HR (95% CI)**	***p***
Sex		
Male	Reference	
Female	1.356 (0.939-1.957)	0.104
Age	0.970 (0.954-0.987)	<0.001
BMI	1.010 (0.955-1.067)	0.721
DM		
No	Reference	
Yes	0.502 (0.265-0.953)	0.035
HTN		
No	Reference	
Yes	0.920 (0.614-1.377)	0.684
Preop. GFR	0.955 (0.944-0.966)	<0.001
Tumor volume	1.005 (1.003-1.006)	<0.001
CNK volume	1.007 (1.002-1.012)	0.003

HR, Hazard ratio; CI, Confidence interval; BMI, Body mass index; DM, Diabetes mellitus; HTN, Hypertension; Preop., Preoperative; GFR, Glomerular filtration rate; CNK, Contralateral normal kidney.
